# Nicotinamide-rich diet protects the heart against ischaemia–reperfusion in mice: A crucial role for cardiac SUR2A

**DOI:** 10.1016/j.phrs.2010.01.008

**Published:** 2010-06

**Authors:** Andriy Sukhodub, Qingyou Du, Sofija Jovanović, Aleksandar Jovanović

**Affiliations:** Division of Medical Sciences, Centre for Cardiovascular and Lung Biology, Ninewells Hospital & Medical School, University of Dundee, Dundee DD1 9SY, UK

**Keywords:** Nicotinamide, Ischaemia, SUR2A, KATP channels

## Abstract

It is a consensus view that a strategy to increase heart resistance to ischaemia–reperfusion is a warranted. Here, based on our previous study, we have hypothesized that a nicotinamide-rich diet could increase myocardial resistance to ischaemia–reperfusion. Therefore, the purpose of this study was to determine whether nicotinamide-rich diet would increase heart resistance to ischaemia–reperfusion and what is the underlying mechanism. Experiments have been done on mice on control and nicotinamide-rich diet (mice were a week on nicotinamide-rich diet) as well as on transgenic mice overexpressing SUR2A (SUR2A mice), a regulatory subunit of cardioprotective ATP-sensitive K^+^ (K_ATP_) channels and their littermate controls (WT). The levels of mRNA in heart tissue were measured by real-time RT-PCR, whole heart and single cell resistance to ischaemia–reperfusion and severe hypoxia was measured by TTC staining and laser confocal microscopy, respectively. Nicotinamide-rich diet significantly decreased the size of myocardial infarction induced by ischaemia–reperfusion (from 42.5 ± 4.6% of the area at risk zone in mice on control diet to 26.8 ± 1.8% in mice on nicotinamide-rich diet, *n* = 6–12, *P* = 0.031). The cardioprotective effect of nicotinamide-rich diet was associated with 11.46 ± 1.22 times (*n* = 6) increased mRNA levels of SUR2A in the heart. HMR1098, a selective inhibitor of the sarcolemmal K_ATP_ channels opening, abolished cardioprotection afforded by nicotinamide-rich diet. Transgenic mice with a sole increase in SUR2A expression had also increased cardiac resistance to ischaemia–reperfusion. We conclude that nicotinamide-rich diet up-regulate SUR2A and increases heart resistance to ischaemia–reperfusion.

## Graphical abstract

**Figure:**
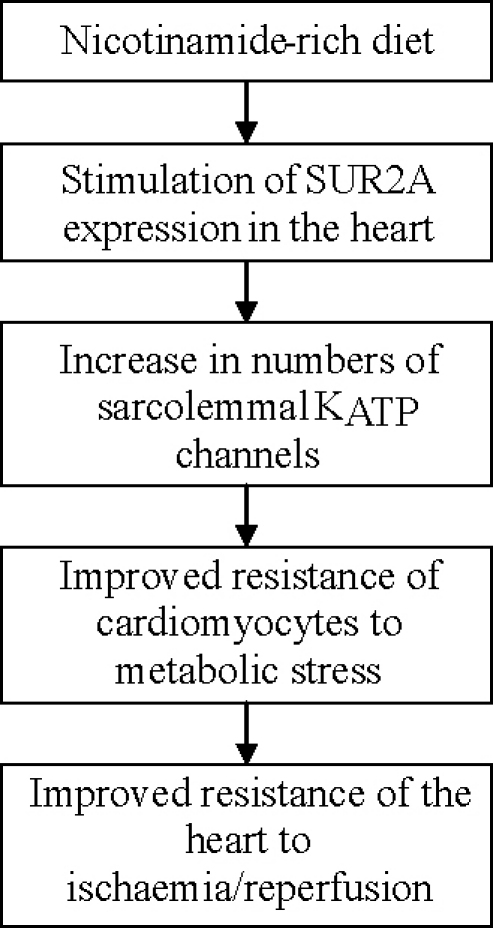

## 1 Introduction

Sarcolemmal K_ATP_ channels were originally discovered in membrane patches excised from ventricular cardiomyocytes (sarcolemmal K_ATP_ channels, [1]). These channels are heteromultimers composed of, at least, two distinct subunits. The pore-forming inwardly rectifying K^+^ channel core, Kir6.2, is primarily responsible for K^+^ permeance, whereas the regulatory subunit, also known as the sulfonylurea receptor, or SUR2A, has been implicated in ligand-dependent channel gating [2]. More recently, it has been suggested that the sarcolemmal K_ATP_ channel protein complex may be composed of more proteins then just Kir6.2 and SUR2A, including Kir6.1 and enzymes regulating intracellular ATP levels and glycolysis [3–8]. Sarcolemmal K_ATP_ channels have been shown to play a crucial role in ischaemic preconditioning (a phenomenon when brief episodes of ischaemia/reperfusion protects the heart against myocardial infarction [9]) and myocardial resistance to ischaemia (reviewed in [10]).

Recent studies have shown that an increase in expression of SUR2A increases the number of sarcolemmal K_ATP_ channels and myocardial resistance to ischaemia/reperfusion [11]. We have found out that female gender, young age or exposure to mild hypoxia is associated with increased levels of SUR2A mRNA as well as numbers of fully assembled K_ATP_ channels and heart resistance to ischaemia/reperfusion [12–14]. When the mechanism of hypoxia-induced increase in SUR2A expression was studied, it was found that an increase in intracellular NAD triggers PI3 kinase signalling pathway leading to activation of SUR2 promoter via c-jun transcription factor [12].

It has been reported that nicotinamide-rich diet increases the intracellular levels of NAD [15]. If increase in NAD up-regulate SUR2A and K_ATP_ channels in cardiac cells, then it is possible that nicotinamide-rich diet would increase myocardial SUR2A/K_ATP_ channels and myocardial resistance to ischaemia. Therefore, we have undertaken this study to examine whether nicotinamide-rich diet would up-regulate SUR2A and increase heart resistance to ischaemia–reperfusion.

## 2 Materials and methods

### 2.1 Nicotinamide-rich diet

C57/BL6J male mice (4–6 weeks old) were fed *ad libidum* with RPM-1 (control diet) or RPM-1 + 0.5 g/kg nicotinamide (nicotinamide-rich diet; Special Diets Services). Each mouse was fed for a week before used for experimentation. All experiments conform to the Home Office Regulations in UK. The experiments have been done under authority of Project Licences 60/3152 and 60/3925.

### 2.2 SUR2A mice

Generation, breeding and genotyping of these mice have previously been described in detail [11]. All experiments conform to the Home Office Regulations in UK. The experiments have been done under authority of Project Licences 60/3152 and 60/3925.

### 2.3 Real-time RT-PCR

Total RNA was extracted from cardiac ventricular tissue of mice using TRIZOL reagent (Invitrogen, Paisley, UK) according to the manufacturer's recommendations. Extracted RNA was further purified with RNeasy Mini Kit (Qiagen, Crawley, UK) according to the manufacturer's instruction. The specific primers for mouse SUR2A, Kir6.2, Kir6.1, SUR1 and SUR2B were described in Ref. [11]. The reverse transcription (RT) reaction was carried out with ImProm-II Reverse Transcriptase (Promega, Southampton, UK). A final volume of 20 μl of RT reaction containing 4 μl of 5× buffer, 3 mM MgCl_2_, 20 U of RNasin^®^ Ribonuclease inhibitor, 1 U of ImProm-II reverse transcriptase, 0.5 mM each of dATP, dCTP, dGTP, and dTTP, 0.5 μg of oligo(dT), and 1 μg of RNA was incubated at 42 °C for 1 h and then inactivated at 70 °C for 15 min. The resulting cDNA was used as a template for real-time PCR. A SYBR Green I system was used for the RT-PCR and the 25 μl reaction mixture contained: 12.5 μl of iQ™ SYBR^®^ Green Supermix (2×), 7.5 nM each primers, 9 μl of ddH_2_O, and 2 μl of cDNA. In principle, the thermal cycling conditions were as follows: an initial denaturation at 95 °C for 3 min, followed by 40 cycles of 10 s of denaturing at 95 °C, 15 s of annealing at 56 °C, and 30 s of extension at 72 °C. The real-time PCR was performed in the same wells of a 96-well plate in the iCycler iQ™ Multicolor Real-Time Detection System (Bio-Rad, Hercules, CA). Data was collected following each cycle and displayed graphically (iCycler iQ™ Real-time Detection System Software, Version 3.0A, BioRad, Hercules, CA). Primers were tested for their ability to produce no signal in negative controls by dimer formation and then with regard to the efficiency of the PCR reaction. Efficiency is evaluated by the slope of the regression curve obtained with several dilutions of the cDNA template. Melting curve analysis tested the specificity of primers. Threshold cycle values, PCR efficiency (examined by serially diluting the template cDNA and performing PCR under these conditions) and PCR specificity (by constructing the melting curve) were determined by the same software. Each mouse cDNA sample was measured at three different quantities, and duplicated at each concentration, the corresponding no-RT mRNA sample was included as a negative control (blank [16]). The calculation of relative mRNA expression was performed as described [17]. The relative expression ratio(R) of SUR2A is calculated using equation $R={\left(E_{\text{K}}\right)}^{\Delta \text{C}{\text{P}}_{\text{K}}(\text{CD-NRD})}/{\left(E_{\text{R}}\right)}^{\Delta \text{C}{\text{P}}_{\text{R}}(\text{CD-NRD})}$ (when the effect of control and nicotinamide-rich diet were assessed) or $R={\left(E_{\text{K}}\right)}^{\Delta \text{C}{\text{P}}_{\text{K}}(\text{WT-TG})}/{\left(E_{\text{R}}\right)}^{\Delta \text{C}{\text{P}}_{\text{R}}(\text{WT-TG})}$ (when wild type and SUR2A transgenic mice were assessed) where *E*_K_ is the real-time PCR efficiency of a SUR2A gene transcript, *E*_R_ is the real-time PCR efficiency of GAPDH (reference) gene, ΔCP_K_ is the crossing point deviation of control-nicotinamide-rich diet (CD-NRD) or wild type-transgene (WT-TG) of SUR2A gene transcript while ΔCP_R_ is the crossing point deviation of control-nicotinamide-rich diet (CD-NRD) or wild type-transgene (WT-TG) of GAPDH gene transcript.

### 2.4 Heart collection and ischaemia–reperfusion injury

The heart collection and ischaemia–reperfusion injury was performed as described in details in our previous papers [18]. In brief, mice were killed by cervical dislocation (according to UK Home Office procedures), and the hearts rapidly removed and placed in ice-cold Tyrode's solution at 4 °C. The aorta was then cannulated and secured using 4–0 silk suture and the hearts were attached to a custom-made Langendorff perfusion apparatus. Hearts were perfused at a constant flow rate of 5 ml/min at 37 °C with oxygenated (95% O_2_, 5% CO_2_; the PO_2_ in perfusate was ∼600 mmHg) Tyrode's solution (in mM: NaCl 136.5, KCl 5.4, CaCl_2_ 1.8, MgCl_2_ 0.53, glucose (Glc) 5.5, HEPES-NaOH 5.5, pH 7.4) for a stabilization period of 30 min. The heart was then subjected to 30 min of ischaemia by placing it into degassed Tyrode's (the solution was degassed with argon for 60 min and the PO_2_ in this solution was ∼20 mmHg) and switching off perfusion. A second 30 min reperfusion with oxygenated Tyrode's followed the ischaemia. When HMR1098 was used, it was present in the Tyrode solution throughout experimental protocol. After reperfusion, hearts were snap-frozen in liquid nitrogen and stored at −80 °C. The frozen heart was divided into approximately 5–6 transverse sections, which were weighed before staining for 1 h in 10% triphenyltetrazolium chloride (TTC) in phosphate buffer saline (PBS; both Sigma–Aldrich, Dorset, UK) at 37 °C. The stain was fixed in 4% paraformaldehyde (Sigma–Aldrich) for 30 min, following which, the tissue was photographed and the area of infarcted tissue measured using Image Analysis software [18]. Infarct sizes were calculated as (*A*_1_ × *W*_1_) + (*A*_2_ × *W*_2_) + (*A*_3_ × *W*_3_) + (*A*_4_ × *W*_4_) + (*A*_5_ × *W*_5_), where *A* is the area of infarct for the slice and *W* is the wt of the respective section [18].

### 2.5 Isolation of single cardiomyocytes

Ventricular cardiomyocytes were dissociated from the mouse using an established enzymatic procedure [19]. In brief, hearts were retrogradely perfused (at 37 °C) with medium 199, followed by Ca^2+^-EGTA-buffered low-Ca^2+^ medium (pCa = 7), and finally low-Ca^2+^ medium containing pronase E (8 mg per 100 ml), proteinase K (1.7 mg per 100 ml), bovine albumin (0.1 g per 100 ml, fraction V) and 200 μM CaCl_2_. Ventricles were cut into fragments in the low-Ca^2+^ medium enriched with 200 μM CaCl_2_. Cells were isolated by stirring the tissue (at 37 °C) in a solution containing pronase E and proteinase K supplemented with collagenase (5 mg per 10 ml). The first aliquot was removed, filtered through a nylon sieve, centrifuged for 60 s (at 300–400 rpm), and washed. Remaining tissue fragments were re-exposed to collagenase, and isolation continued for 2–3 such cycles.

### 2.6 Experimental protocol of severe cellular hypoxia

Severe hypoxia of isolated cardiomyocytes has been performed as described [20]. Thus, cardiomyocytes were placed into Tyrode's solution (in mM: NaCl 136.5, KCl 5.4, CaCl_2_ 1.8, MgCl_2_ 0.53, glucose 5.5, HEPES-NaOH 5.5, pH 7.4), plated out on glass coverslips and paced to beat by field stimulation (parameters of the stimulation: 5–20 mV depending on cellular threshold, 5 ms, 1 Hz). Beating cardiomyocytes were perfused with Tyrode solution at a rate of 3 ml/min and, under these conditions, the partial pressure of O_2_ (PO_2_) in perfusate was 140 mmHg. To induce severe hypoxia, Tyrode solution was bubbled with 100% argon (PO_2_ = 20 mmHg was achieved in the solution surrounding cardiomyocytes; to achieve this level of hypoxia, solution was degassed for 60 min). When HMR1098 was used, it was present in the Tyrode solution throughout experimental protocol. To clearly visualize cells we have loaded them di-8-ANEPPS according to the manufacturer's instruction (Invitrogen, Paisley, UK). Cells were imaged using laser confocal microscopy in line-scan mode (LSM-510, Zeiss, Göttingen, Germany). Fluorescence was detected/imaged at 488 nM excitation wavelength and emission was captured at >505 nM. The moment of cell death was defined as the point when the cell has become rounded (ratio of diameters <2 [21]).

### 2.7 Statistical analysis

Data are presented as mean ± SEM, with *n* representing the number of analysed mice or cells. Mean values were compared by the ANOVA followed by Student's *t*-test, Mann–Whitney rank sum test or by Chi-square test where appropriate using SigmaStat program (Jandel Scientific, Chicago, IL). *P* < 0.05 was considered statistically significant.

## 3 Results

### 3.1 SUR2A mRNA levels in mice on control and nicotinamide-rich diet

**Fig. 1:**
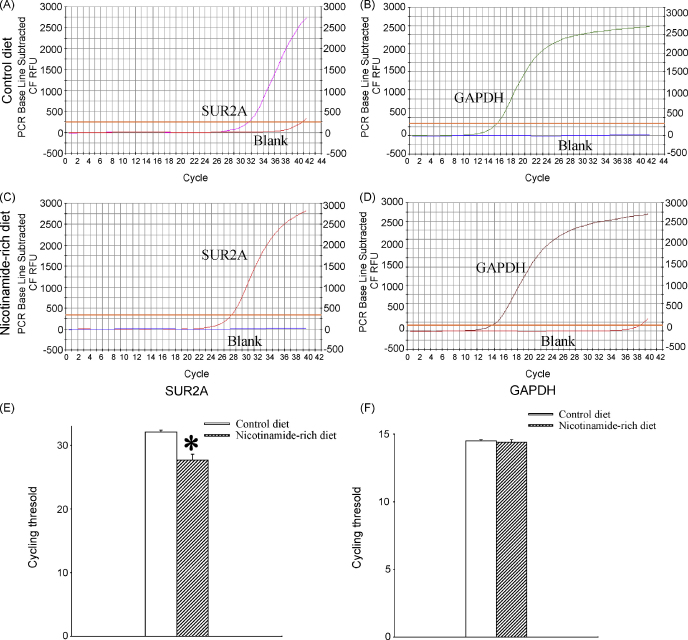
Expression of SUR2A in hearts of mice on control and nicotinamide-rich diet. Representative progress curves for the real-time PCR amplification of SUR2A (A and C) and GAPDH (B and D) cDNA from mice on control or nicotinamide-rich diet (as labelled on the figure) and a corresponding bar graphs (E and F). Each bar represents mean ± standard error of the mean (*n* = 6 for each). *P < 0.05.

We have analysed levels of SUR2A mRNA in mice that were fed with control and nicotinamide-rich diet. Real-time RT-PCR revealed that nicotinamide-rich diet significantly increased the levels of SUR2A mRNA in the heart as the threshold cycle for mice on control and nicotinamide-rich diet was 32.1 ± 0.3 and 27.7 ± 0.9, respectively (*n* = 6 for each, *P* < 0.01, Fig. 1). On the other hand, no significant difference was found in GAPDH mRNA levels (Fig. 1; threshold cycle was 14.5 ± 0.1 for mice on control and 14.4 ± 0.2 on nicotinamide-rich diet, *n* = 6 for each, *P* = 0.85). It was calculated that mice on nicotinamide-rich diet had 11.46 ± 1.22 times more SUR2A mRNA in the heart than mice on control diet (Fig. 1). Nicotinamide-rich diet did not alter the expression of Kir6.2 and Kir6.1 (Kir6.1: threshold cycle for was 21.1 ± 0.4 for mice on control and 21.6 ± 0.3 for mice on nicotinamide-rich diet, *P* = 0.23, *n* = 6; Kir6.2: threshold cycle for was 22.7 ± 1.7 for mice on control and 23.2 ± 0.4 for mice on nicotinamide-rich diet, *P* = 0.25, *n* = 4–6).

### 3.2 Nicotinamide-rich diet increases heart resistance to ischaemia–reperfusion

**Fig. 2:**
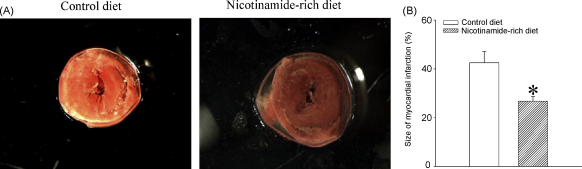
Resistance of hearts to ischaemia–reperfusion from mice on control and nicotinamide-rich diet. (A) Typical photographs of myocardial slices from mice exposed to ischaemia–reperfusion under depicted conditions. Infarcted areas are pale/grey while viable myocardium is dark/red. (B) Bar graphs depict myocardial infarct size expressed as a percentage of area at risk zone (*n* = 6–12). *P < 0.05.

Ischaemia–reperfusion induced myocardial infarction in mice on control diet that was 42.5 ± 4.6% of the area at risk zone (*n* = 12, Fig. 2). The size of myocardial infarction was significantly smaller in mice on nicotinamide-rich diet (26.8 ± 1.8%, *n* = 6, *P* = 0.031, Fig. 2).

### 3.3 A sole increase in SUR2A mimic the cardioprotective effect of nicotinamide-rich diet

**Fig. 3:**
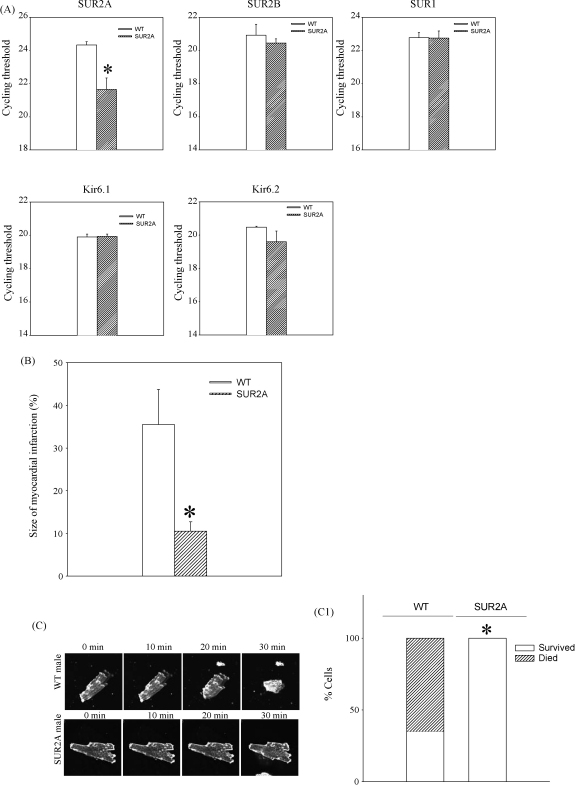
A sole increase in expression mimics the cardioprotective effect of nicotinamide-rich diet. (A) Real-time RT-PCR of K_ATP_ channel subunits in the heart. Bar graphs represent cycling thresholds of the real-time RT-PCR progress curves of K_ATP_ channel-forming subunits. Each bar represents mean ± SEM (*n* = 6 for each), *P < 0.05. (B) Bar graphs depict myocardial infarct size expressed as a percentage of area at risk zone (*n* = 5–9), *P < 0.05. (C) Laser confocal images of cardiac cells from wild type (WT) and transgenic (SUR2A) mice exposed to hypoxia (magnification was 40×). Bar graph depicts percentage of cells that died/survived 30 min-long hypoxia, *n* = 14–28, *P < 0.01.

In addition to up-regulating SUR2A expression, nicotinamide-rich diet might have other effects. If nicotinamide-rich diet is cardioprotective due to increased SUR2A expression, then a sole increase in SUR2A would be sufficient to mimic this effect of nicotinamide-rich diet. To determine whether increased expression of SUR2A is responsible for the increased myocardial resistance to ischaemia–reperfusion, we have used SUR2A-overexpressing transgenic mice. It has been already reported that a sole increase in SUR2A increases myocardial resistance to ischaemia–reperfusion in mice, but whether this is the case in male mice alone has not yet been assessed. Therefore, we have assessed here myocardial resistance exclusively of male mice overexpressing SUR2A. Real-time RT-PCR has confirmed that transgenic intervention has significantly increased mRNA levels of SUR2A (cycling threshold was 24.3 ± 0.2 in wild type and 21.6 ± 0.7 in transgenics, *n* = 6 for each, *P* < 0.01, Fig. 3). No statistically significant difference was observed in mRNA levels of other K_ATP_ channel-forming subunits (Fig. 3) as well as GAPDH expression (cycling threshold was 18.9 ± 0.5 in wild type and 19.1 ± 0.3 in transgenics, *n* = 6 for each, *P* = 0.74). Transgenic mice had 6.54 ± 0.41 times more SUR2A mRNA then the wild type in the heart.

In wild type, the size of myocardial infarction followed by ischaemia–reperfusion was 35.5 ± 8.2% of the area at risk zone (*n* = 9, Fig. 3). The size of myocardial infarction was significantly smaller in transgenic mice (10.5 ± 2.2% of the area at risk zone, *n* = 5, *P* = 0.036, Fig. 3).

To provide further evidence that improvement in myocardial resistance to ischaemia/reperfusion is due to the effect that SUR2A has on cardiomyocytes, we have tested the effect of severe hypoxia in single cardiomyocytes from the two phenotypes. When exposed to severe single cell hypoxia, 9 out of 14 cells from five wild type males have died during first 30 min of severe hypoxia (Fig. 3). On the other hand, all 28 tested cells from six male transgenic mice have survived 30 min-long severe hypoxia (Fig. 3).

### 3.4 HMR1098, a selective antagonist of sarcolemmal K_ATP_ channels, abolishes cardioprotection afforded by nicotinamide

**Fig. 4:**
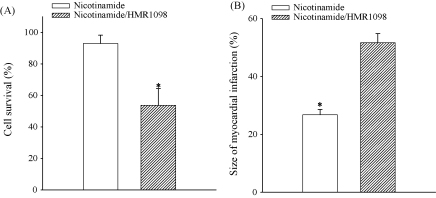
HMR1098, a selective antagonist of sarcolemmal K_ATP_ channels, abolishes cardioprotection afforded by nicotinamide-rich diet. (A) Bar graph depicting percentage of cells from mice on nicotinamide-rich diet in the absence (nicotinamide) or presence of 30 μM HMR 1098 (nicotinamide/HMR1098) that died following 30 min-long hypoxia, *n* = 1698–1863, *P < 0.01. (B) Bar graphs depicting myocardial infarct size in mice on nicotinamide-rich diet in the absence (nicotinamide) or presence of 30 μM HMR 1098 (nicotinamide/HMR1098; *n* = 5–6), *P < 0.01.

Apart on regulating expression of SUR2A, nicotinamide could regulate the expression of other genes as well. It has been previously shown that inhibition of the activation of sarcolemmal K_ATP_ channels inhibit cardioprotection afforded by increased sarcolemmal K_ATP_ channels [11]. Therefore, if the cardioprotective effect of nicotinamide *per os* is mediated by up-regulation of SUR2A, then HMR1098, a selective antagonist of sarcolemmal K_ATP_ channels should block nicotinamide-mediated cardioprotection. Under control conditions 92.9 ± 5.3% of cells from mice on nicotinamide *per os* have survived 30 min-long severe hypoxia (1698 cells from three mice were analysed). In contrast, only 53.7 ± 10.5% of cardiac cells from the same animals survived 30 min of severe hypoxia (1863 cells from three mice were analysed) in the presence of HMR1098 (30 μM). The difference between cell survival in the absence and presence of HMR1098 was statistically significant (Fig. 4, *P* < 0.001). Similar results were obtained at the whole heart level, where the size of myocardial infarction in response to ischaemia–reperfusion in the presence of HMR1098 (30 μM) was 51.7 ± 3.1% (*n* = 5), which was significantly different to this value obtained in the absence of HMR1098 (26.8 ± 1.8%, *n* = 6, *P* < 0.001, Fig. 4). The size of myocardial infarction in response to ischaemia–reperfusion was not different between mice on control diet and mice on nicotinamide-rich diet when the ischaemia–reperfusion was induced in the presence of HMR1098 (30 μM, *P* = 0.24; *n* = 5–6, Fig. 4).

## 4 Discussion

Here, we have demonstrated that nicotinamide-rich diet protect the heart against ischaemia–reperfusion by increasing the expression of SUR2A, a regulatory subunit of sarcolemmal K_ATP_ channel.

It has been shown that conditions associated with increased expression of SUR2A results in increase in myocardial resistance to ischaemia–reperfusion, which seems to be due to increased numbers of sarcolemmal K_ATP_ channels [11–14]. It has been suggested that up-regulation of SUR2A is sufficient to increase numbers of sarcolemmal K_ATP_ channels as this subunit seems to be the least expressed K_ATP_ channel-forming protein making intracellular SUR2A level a rate-limiting step in assembling fully functional K_ATP_ channels [11]. The activation of these channels shortens action membrane potential during ischaemia–reperfusion preventing influx of Ca^2+^ and Ca^2+^ overload, which is the main cause of cell death under this condition. In addition to that, K_ATP_ channels also seem to produce ATP during ischaemia and promote cell survival by maintaining physiological levels of subsarcolemmal ATP [22,23]. The ATP producing property of K_ATP_ channels is probably due to the presence of creatine kinase and glycolytic enzymes in sarcolemmal K_ATP_ channel protein complex *in vivo* [5–8]. The dual mechanism of cytoprotection by K_ATP_ channels [22,23] can probably explain why an increase in number of sarcolemmal K_ATP_ channels seems to be a more efficient in protecting the heart against ischaemia, than just their activation [24].

It has been reported that an increase in intracellular NAD stimulate SUR2A expression by activating PI3-kinase signalling pathway that stimulates SUR2 promoter via c-jun transcription factor [12]. Further experiments have demonstrated that increase in intracellular NAD triggers signalling pathway that up-regulate SUR2A. Nicotinamide-rich diet is known to increase levels of NAD in many tissues, although this has not been specifically reported for the heart [15]. Here, we have shown that nicotinamide-rich diet increases SUR2A mRNA in the heart, which supported our hypothesis, based on results from previous studies [12,15], that nicotinamide-rich diet might up-regulate SUR2A by increasing intracellular NAD and activating SUR2 promoter.

Nicotinamide-rich diet has significantly increased heart resistance to ischaemia–reperfusion and this has never been shown before. In contrast to niacin, nicotinamide does not affect cholesterol levels and has no use in treatment of dyslipidemia (reviewed in [25]) and *ex vivo* design of our experiments has demonstrated that nicotinamide-rich dieat increases cardiac resistance to ischaemia–reperfusion by direct action on the myocardium. As the cardioprotection was associated with increase in SUR2A expression, it was feasible to conclude that increase in SUR2A levels is the mechanism underlying cardioprotection afforded by nicotinamide-rich diet.

However, nicotinamide-rich diet certainly has other effects in addition to SUR2A up-regulation (reviewed in [26]). It was therefore possible that increase in SUR2A was just an epiphenomenon that actually was not responsible for increased heart resistance to ischaemia–reperfusion. Therefore, we have used transgenic mice with solely increased expression of SUR2A devoid of any SUR2A-independent effects that nicotinamide might have. Real-time RT-PCR has confirmed that that transgenic mouse had increased expression of SUR2A while the expression of other K_ATP_ channel-forming subunits has remained intact. It has been previously suggested that SUR2A phenotype acquire resistance to myocardial ischaemia–reperfusion, but this has been shown on mixed gender population of mice [11]. Here, we have tested transgenic mice and littermate controls that were gender and age-matched with mice on control and nicotinamide-rich diet. The obtained results have shown that a sole increase in SUR2A expression is sufficient to increase myocardial resistance to ischaemia–reperfusion. Finally, the fact that HMR1098, a selective antagonist of the sarcolemmal K_ATP_ channels opening, abolished nicotinamide-induced cardioprotection on both single cell and whole heart levels provide a direct link between the increase in SUR2A, sarcolemmal K_ATP_ channels and cardioprotection afforded by nicotinamide. It has been previously shown that conditions with up-regulated SUR2A is associated with increased numbers of sarcolemmal K_ATP_ channels and increased cardiac resistance to metabolic stress. In addition, SUR2A-mediated increase in heart resistance to ischaemia–reperfusion was, without any exceptions, sensitive to HMR1098 [11–14]. Thus, the effect of HMR1098 on nicotinamide-induced cardioprotection is consistent with the notion that this cardioprotection is mediated by up-regulation of SUR2A. The efficiency of HMR1098 in blocking nicotinamide-mediated cardioprotection would imply that stimulation of SUR2A expression is the main mechanism of cardioprotection afforded by nicotinamide.

Nicotinamide *per os* has been used so far to treat pellagra and *acne vulgaris* and it is known as a very safe drug [27]. Here, for the first time we have shown that this compound could be used to treat heart ischemia, which could be a perfect adjunct to current therapeutic strategies against ischaemic heart diseases, based on restitution of blood flow to the heart and decrease of myocardial metabolic demand (see also [24]).
